# Supplementary material for: Application of an analytical framework for multivariate mediation analysis of environmental data
Source: Nat Commun. 2020 Nov 6;11:5624. doi: 10.1038/s41467-020-19335-2 (PMC7648785; doi:10.1038/s41467-020-19335-2)
Supplement: Supplementary file 1 — Supplementary Information [file 41467_2020_19335_MOESM1_ESM.pdf]

Supplemental Table 1. Comparison of subset sample to LIFECODES larger sample

	Subset (N=161)	Overall LIFECODES (N=482)
**Sample Characteristics**	**Median (IQR)**	**Median IQR**
Age (years)	32.8 (4.8)	32.7 (6.7)
Gestational age at delivery (weeks)	38.7 (2.0)	39 (2.2)
	**Count (percent)**	**Count (percent)**
Overall preterm birth		
Case	52 (32.3%)	130 (27%)
Control	109 (67.7%)	352 (73%)
Spontaneous preterm birth		
Case	30 (21.6%)	75 (17.6%)
Control	109 (78.4%)	352 (82.4%)
Initial visit BMI (median 10 weeks gestation)		
<25 kg/m^2	83 (51.6%)	250 (51.9%)
25-29.9 kg/m^2	46 (28.7%)	126 (26.1%)
≥30 kg/m^2	32 (19.7%)	102 (21.2%)
Missing	-	4 (0.8%)
Race		
White	106 (65.7%)	282 (58.5%)
Black	17 (10.9%)	77 (16.0%)
Other	38 (23.4%)	123 (25.5%)
Education level		
High school degree	18 (11.5%)	68 (14.1%)
Technical school	15 (9.3%)	77 (16.0%)
Junior college or some college	56 (34.6%)	139 (28.8%)
College graduate	72 (44.6%)	187 (38.8%)
Missing	-	11 (2.3%)
Insurance		
Private/HMO/Self-pay	146 (90.7%)	385 (79.9%)
Medicaid/SSI/MassHealth	15 (9.3%)	85 (17.6%)
Missing	-	12 (2.5%)
Tobacco use		
No smoking during pregnancy	152 (94.6%)	445 (92.3%)
Smoked during pregnancy	9 (5.4%)	31 (6.4%)
Missing	-	6 (1.2%)
Alcohol use		
No alcohol use during pregnancy	153 (95.0%)	452 (93.8%)
Alcohol use during pregnancy	8 (5.0%)	20 (4.1%)
Missing	-	10 (2.1%)
Fetal sex		
Female	75 (46.3%)	213 (44.4%)
Male	86 (53.7%)	267 (55.6%)

Supplemental Table 2. Correlation Matrix Between Toxicant Risk Scores

	Phthalate Risk Score	Phenol Risk Score	PAH Risk Score	Metal Risk Score
Phthalate Risk Score	1	-0.15	-0.23	0.03
Phenol Risk Score	-0.15	1	0.26	-0.04
PAH Risk Score	-0.23	0.26	1	0.04
Metal Risk Score	0.03	-0.04	0.04	1

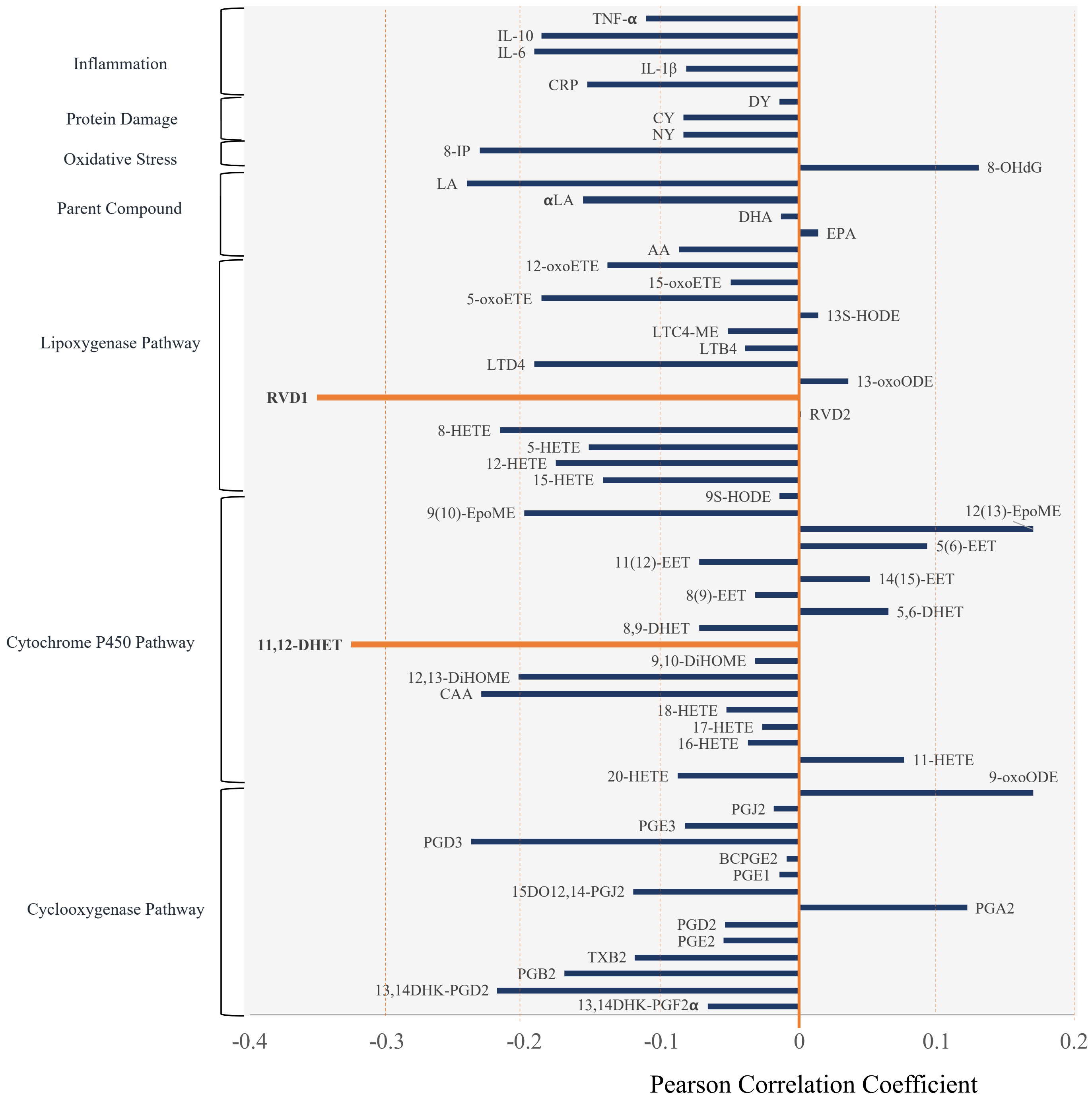

Supplemental Figure 1. Bar chart of correlation coefficients between the first direction of mediation for phthalate risk score and individual mediators. Estimates are weighted for inverse probability weights. Orange bars represent correlation coefficients exceeding |0.3|.

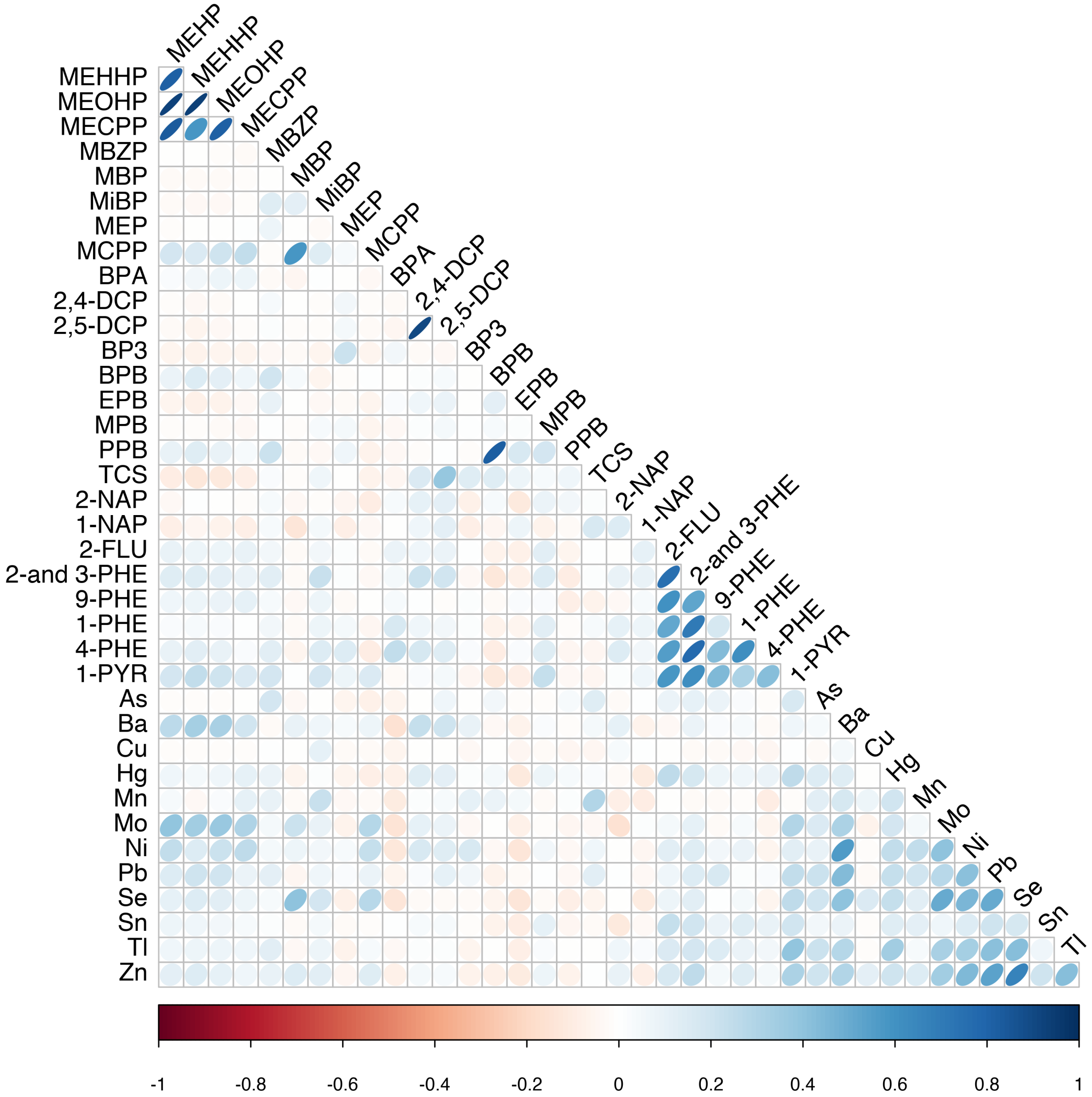

Supplemental Figure 2. Correlation matrix of individual environmental toxicants. Estimates are based on specific gravity adjusted concentrations and weighted for inverse probability weights.

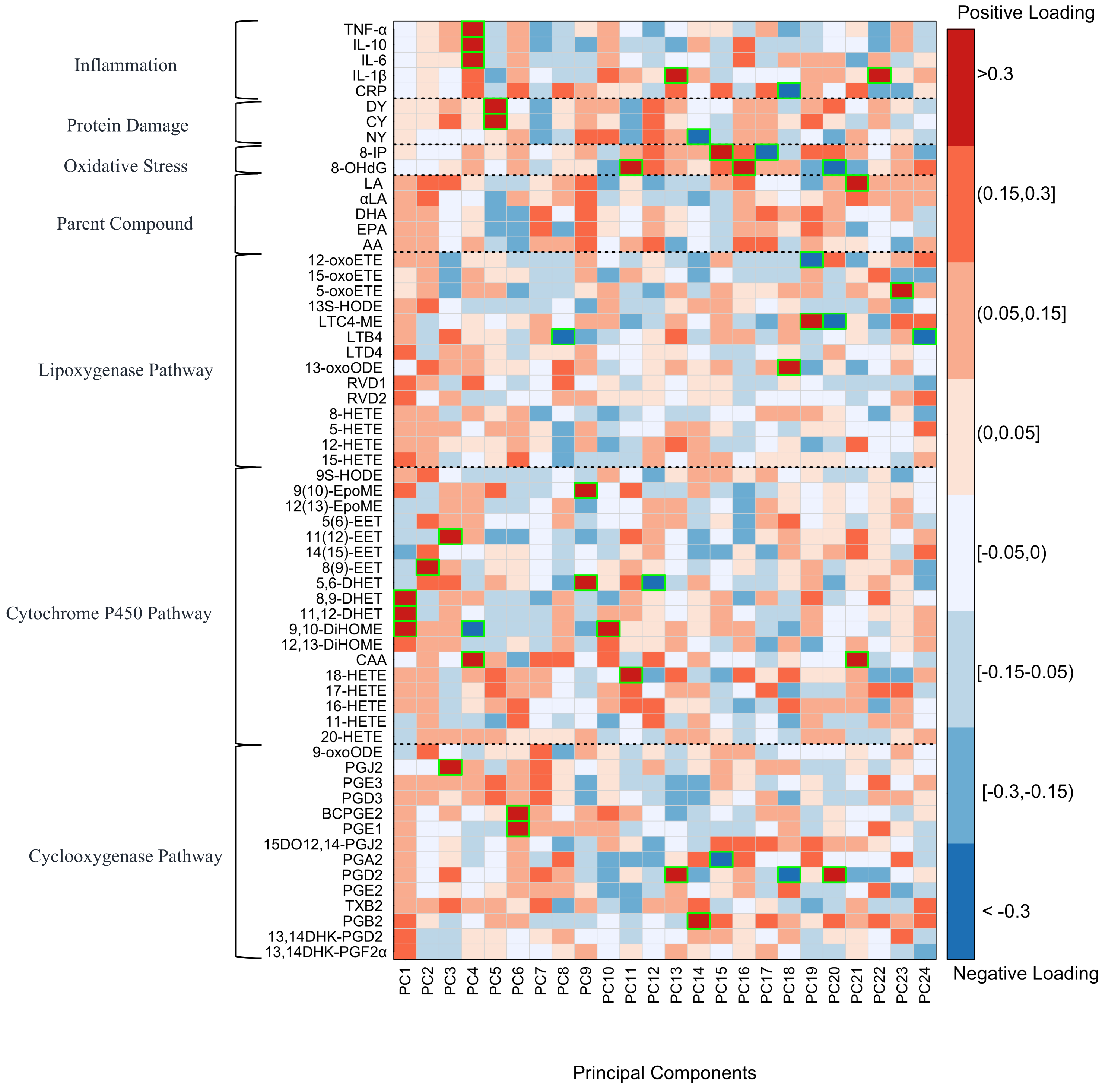

Supplemental Figure 3. Heat map of loading values for individual mediators relative to individual principal components estimated from sparse principal component based mediation analysis. Red and blue grids indicate positive and negative loadings, respectively. Color intensities represent the magnitude of loading, i.e. darker grids indicate greater loading. Mediators with loadings exceeding |0.3| are labeled by green squares.

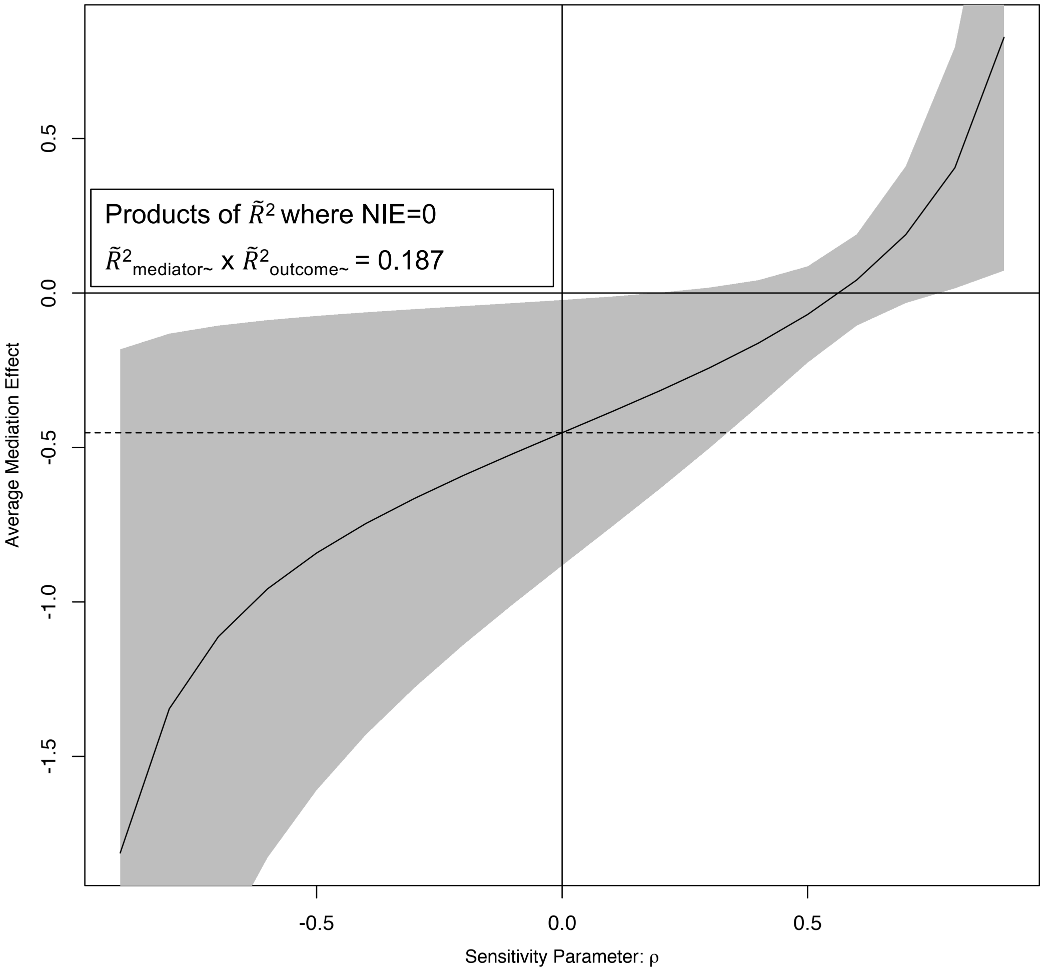

Supplemental Figure 4. Fitted plot of the sensitivity parameter ρ (x-axis) and average causal mediation effect (y-axis) estimated from the *medsens* function in the *mediate* package (version 4.5.0). The dashed horizontal line represents the estimated average causal mediation effect when ρ equals to zero. Legend reports the product of $\tilde{R}^2$ when the mediation effect equals zero. Data are presented as fitted regression estimates and 95% confidence intervals (instantaneous fitted estimate * ±1.96(standard error[fitted estimate])

Abbreviation: Natural indirect effect (NIE)

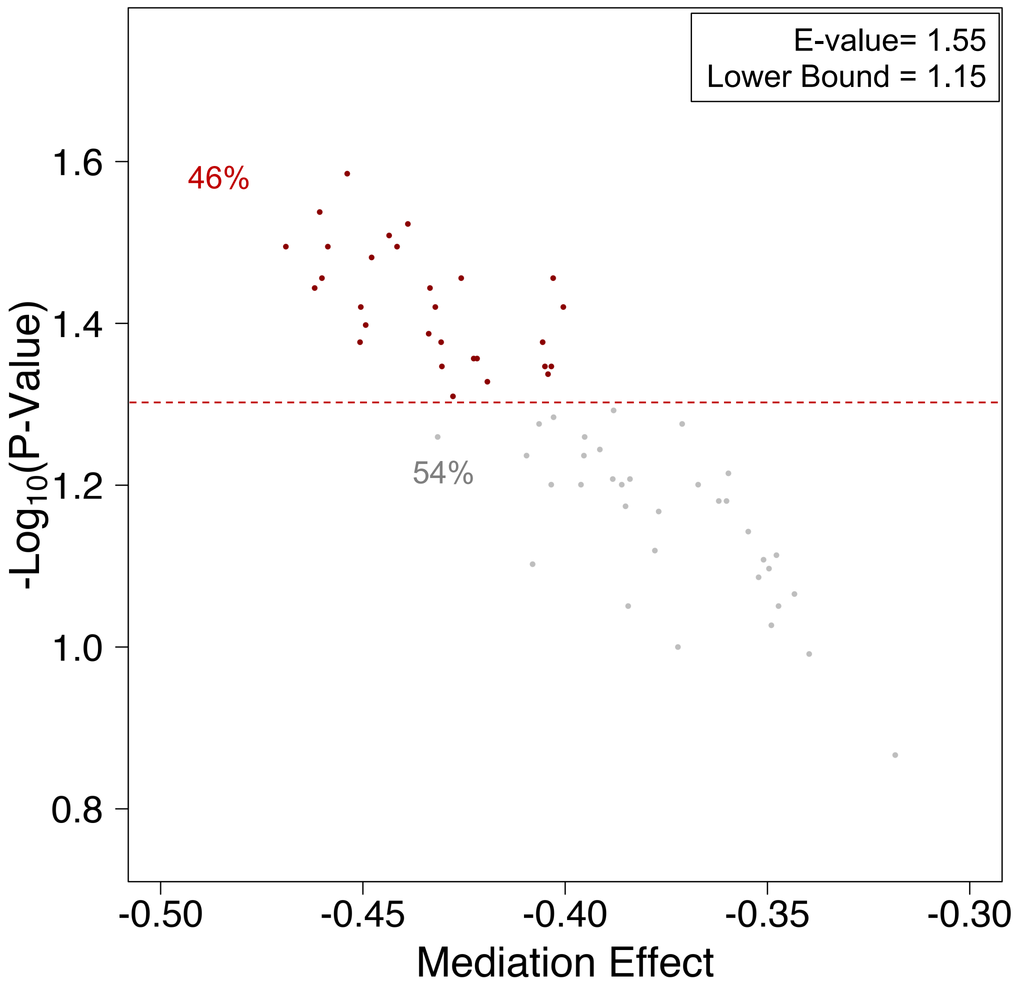

Supplemental Figure 5. Scatter plot of mediation effects (x-axis) and -Log_{10}(P-value) (y-axis) for all possible combination of six covariates (n_{combo}=63): specific gravity, maternal age, education, race, maternal BMI at initial study visit, and health insurance provider. Horizontal dashed red line indicates p-value <0.05. Combinations with p-value <0.05 are highlighted in red, while combinations with p-value> 0.05 are highlighted in grey. E-value estimation calculated for the final model in the main analysis, using the approximation of risk ratio transformation of standardized mediation effect estimate.

Section 1. Notations, definitions, and assumptions.

Suppose our analysis is based on a study of n subjects, and for each subject i, $i = 1, \ldots, n$, we collect data on the exposure A_i, q candidate mediators $\boldsymbol{M_i} = (M_i^{(1)}, M_i^{(2)}, \ldots, M_i^{(q)})$, the outcome Y_i, r covariates $\boldsymbol{C_i} = (C_i^{(1)}, C_i^{(2)}, \ldots, C_i^{(r)})$. Indeed, to formally define causal effects and draw causal conclusions, we adopt a counterfactual framework for causal mediation analysis in the presence of multiple mediators. We define $\boldsymbol{M_i}(a) = \left(M_i^{(1)}(a), M_i^{(2)}(a), \ldots, M_i^{(q)}(a)\right)$ as the i-th subject's counterfactual value of the q mediators if they received exposure a, and define $Y_i(a, \boldsymbol{m})$ as this subject's counterfactual outcome under exposure level at a and mediators at $\boldsymbol{m} = (m^{(1)}, m^{(2)}, \ldots, m^{(q)})$. With these notations, we can formally define the direct effect and effect mediated through the multiple mediators, i.e. indirect effect. The natural direct effect (NDE) is defined as $Y_i\left(a, \boldsymbol{M_i}(a^*)\right) - Y_i(a^*, \boldsymbol{M_i}(a^*))$, which is the change in the counterfactual outcomes when exposure changes from a^*(the reference level) to a while hypothetically controlling mediators at the level that they would have naturally been with exposure a^*. The natural indirect effect (NIE) is defined as $Y_i\left(a, \boldsymbol{M_i}(a)\right) - Y_i(a, \boldsymbol{M_i}(a^*))$, the change in counterfactual outcomes when mediators change from $\boldsymbol{M_i}(a^*)$ to $\boldsymbol{M_i}(a)$ while fixing the exposure at a. The total effect (TE) can then be expressed as the summation of the NDE and the NIE: $Y_i\left(a, \boldsymbol{M_i}(a)\right) - Y_i(a^*, \boldsymbol{M_i}(a^*)) = Y_i\left(a, \boldsymbol{M_i}(a)\right) - Y_i(a, \boldsymbol{M_i}(a^*)) + Y_i\left(a, \boldsymbol{M_i}(a^*)\right) - Y_i(a^*, \boldsymbol{M_i}(a^*)) =$ NDE + NIE.

The counterfactual variables used to define causal effects are not necessarily observed, but the identification of causal effects must be based on observed data. Therefore, further assumptions regarding the confounders are needed for the identification and interpretation of causal effects[1]. We will use $A \perp B \mid C$ to denote that A is independent of B conditional on C. To estimate the average NDE and NIE from observed data, the following identifiability assumptions are required:

(1) $Y_i(a, \boldsymbol{m}) \perp A_i \mid \boldsymbol{C_i}$, that is, no unmeasured confounding for exposure-outcome relationship;

(2) $Y_i(a, \boldsymbol{m}) \perp \boldsymbol{M_i} \mid \{\boldsymbol{C_i}, A_i\}$, that is, no unmeasured confounding for any of mediator-outcome relationship after controlling for the exposure;

(3) $\boldsymbol{M_i}(a) \perp A_i \mid \boldsymbol{C_i}$, that is, no unmeasured confounding for the exposure effect on all the mediators;

(4) $Y_i(a, \boldsymbol{m}) \perp \boldsymbol{M_i}(a^*) \mid \boldsymbol{C_i}$, that is, no downstream effect of the exposure that confounds any mediator-outcome relationship.

The above four assumptions are required to hold with respect to the whole set of mediators. Finally, as in all mediation analysis, the temporal ordering assumption also needs to be satisfied, i.e., the exposure precedes the mediators, and the mediators precede the outcome.

First, we note that the identifiability assumptions cannot be verified empirically from the observed data[2], and we can only justify our selection of covariates based on scientific knowledge. For both the outcome and mediator model, we adjust for specific gravity, maternal age, race, BMI at initial study visit, education level and health insurance provider as confounders. For assumption (1), we believe that those available covariates are natural to control for exposure-outcome confounders based on existing domain-specific literature[3]. For assumption (2), within each exposure level, those covariates are also natural to control for confounders that are associated with both endogenous biomarkers and gestational age at delivery[4]. For assumption (3), we included all the important potential exposure-mediator confounders as in another similar study[5]. Assumption (4) is usually a challenging condition to justify as it simultaneously involves counterfactuals with $A_i = a$ and $A_i = a^*$, one of which will not be observed in real data. The influence of violating the above identifiability assumptions can be assessed using sensitivity analysis, which has been well-developed for the single mediator setting[6], and additional work is required to extend that approach to the multiple-mediator setting.

Regarding the temporal assumptions, in the LIFECODES birth cohort, participants provided biological specimens (urine and blood) at a clinic visit occurring between 23.1 and 28.9 weeks gestation. All exposure analytes were measured in urine samples, and the potential mediators, a large panel of 53 eicosanoids and lipid metabolites, were measured in plasma samples. The outcome of interest, gestational age of the newborn was recorded at delivery. Therefore, the exposure and mediators comes earlier than the outcome variable. While it is difficult to disentangle the temporal ordering between the exposure and mediators' measurements, our conceptual model supports the statistical model. Exposure to toxicants (e.g. phthalates, toxic heavy metals) may disturb receptor activity and induce their responses, which could affect the signaling molecules related to inflammation and metabolism. Biomarkers of inflammation and oxidative stress have been shown to be associated with the risk of preterm birth.

References

1. Vanderweele, T. J. & Vansteelandt, S. Mediation Analysis with Multiple Mediators. *Epidemiologic Methods* **2,** 95–115 (2014).
2. Rubin, D. B. & Little, R. *Statistical Analysis with Missing Data, Vol. 793.* (2019).
3. Ferguson, K. K., McElrath, T. F., Ko, Y.-A., Mukherjee, B. & Meeker, J. D. Variability in urinary phthalate metabolite levels across pregnancy and sensitive windows of exposure for the risk of preterm birth. *Environment International* **70,** 118–124 (2014).
4. Aung, M. T. *et al.* Prediction and associations of preterm birth and its subtypes with eicosanoid enzymatic pathways and inflammatory markers. *Scientific Reports* 1–17 (2019). doi:10.1038/s41598-019-53448-z
5. Ferguson, K. K. *et al.* Mediation of the Relationship between Maternal Phthalate Exposure and Preterm Birth by Oxidative Stress with Repeated Measurements across Pregnancy. *Environ Health Perspect* **125,** 488–494 (2017).
6. Imai, K., Keele, L. & Yamamoto, T. Identification, Inference and Sensitivity Analysis for Causal Mediation Effects. *Statist. Sci.* **25,** 51–71 (2010).
