# Supplementary material for: Application of an analytical framework for multivariate mediation analysis of environmental data
Source: Nat Commun. 2020 Nov 6;11:5624. doi: 10.1038/s41467-020-19335-2 (PMC7648785; doi:10.1038/s41467-020-19335-2)
Supplement: Supplementary file 11 — Description of Additional Supplementary Files [file 41467_2020_19335_MOESM11_ESM.pdf]

Description of Supplementary Data Files

Title: Supplementary Data 1A
Description: Pairwise mediation estimates for overall preterm birth. Models adjusted for specific gravity, maternal age, race, education, health insurance, and BMI at initial study visit. All p-values estimated using a two-sided test for t-statistics from linear regression models. Adjustments for multiple comparisons were accounted for by calculating q-values.

Title: Supplementary Data 1B
Description: Pairwise mediation estimates for spontaneous preterm birth. Models adjusted for specific gravity, maternal age, race, education, health insurance, and BMI at initial study visit. All p-values estimated using a two-sided test for t-statistics from linear regression models. Adjustments for multiple comparisons were accounted for by calculating q-values.

Title: Supplementary Data 1C
Description: Pairwise mediation estimates for gestational age at delivery. Models adjusted for specific gravity, maternal age, race, education, health insurance, and BMI at initial study visit. All p-values estimated using a two-sided test for t-statistics from linear regression models. Adjustments for multiple comparisons were accounted for by calculating q-values.

Title: Supplementary Data 2
Description: Posterior inclusion probabilities estimated from Bayesian shrinkage mediation analysis

Title: Supplementary Data 3
Description: Mediation estimates of individual toxicants and reduced mediator matrix in association with gestational age at delivery. Models adjusted for specific gravity, maternal age, race, education, health insurance, and BMI at initial study visit. All p-values estimated using a two-sided test for t-statistics from linear regression models. Adjustments for multiple comparisons were accounted for by calculating q-values.

Title: Supplementary Data 4
Description: Correlation coefficients between individual exposures and mediators

Title: Supplementary Data 5
Description: Mediation estimates of environmental risk scores and individual mediators in association with gestational age at delivery. Models adjusted for specific gravity, maternal age, race, education, health insurance, and BMI at initial study visit. All p-values estimated using a two-sided test for t-statistics from linear regression models. Adjustments for multiple comparisons were accounted for by calculating q-values.

Title: Supplementary Data 6
Description: Mediation estimates for reduced exposure and mediator matrices in association with gestational age at delivery. Models adjusted for specific gravity, maternal age, race, education, health insurance, and BMI at initial study visit. All p-values estimated using a two-sided test for t-statistics from linear regression models. Adjustments for multiple comparisons were accounted for by calculating q-values.

Title: Supplementary Data 7
Description: Mediation estimates for phthalate risk score and sparse principal components in association with gestational age at delivery. Models adjusted for specific gravity, maternal age, race, education, health insurance, and BMI at initial study visit. All p-values estimated using a two-sided test for t-statistics from linear regression models. Adjustments for multiple comparisons not necessary in this setting given that only one model was built for sparse principal components analysis.
